# Commentary: Association of Sarcopenia with Toxicity and Survival in Postoperative Recurrent Esophageal Squamous Cell Carcinoma Patients Receiving Chemoradiotherapy

**DOI:** 10.3389/fonc.2021.806858

**Published:** 2022-01-05

**Authors:** Zumrut Bahat, Gulistan Bahat

**Affiliations:** ^1^ Department of Radiation Oncology, Faculty of Medicine, Karadeniz Technical University, Trabzon, Turkey; ^2^ Department of Internal Medicine, Division of Geriatrics, Istanbul Medical School, Istanbul University, Istanbul, Turkey

**Keywords:** sarcopenia, cancer, prognosis, radiotherapy, muscle strength, function

## Introduction

In their valuable article, Xu et al. examined the prevalence of sarcopenia in postoperative recurrent esophageal squamous cell carcinoma (ESCC) patients receiving chemoradiotherapy (CRT). The authors studied the association of sarcopenia (S) with treatment-related toxicity and prognosis (overall survival, OS) as well (1). They included 184 patients over a 2-year period and assessed skeletal muscle area at the third lumbar vertebra. They adjusted skeletal muscle area by height^2^, defining low skeletal muscle mass as lower than 47.24/cm^2^/m^2^ in men and 36.92/cm^2^/m^2^ in women. They reported that S was present in about half (51.1%) of the patients. Patients with S showed decreased OS (hazard ratio = 1.729, 95% confidence interval 1.231–2.428, *p* = 0.002) in the multivariate analysis. In the multivariate model, the presence of sarcopenia was the most significant independent prognostic factor of poor OS (*p* = 0.002) followed by worse KPS and advanced tumor stage. While they reported that rates of grade 3–4 toxicities were higher in sarcopenic participants in univariate analyses, they did not study if S was an independent factor for treatment-related toxicities in multivariate analyses.

## Comment on the Findings and Discussion

In this study, S was considered as the presence of low muscle mass. S was defined as an age-related loss of skeletal muscle mass (SMM) at its first conceptualization. Yet, many studies reported that low skeletal muscle function is related to survival and other adverse outcomes as well, even more than the LMM (2, 3). Moreover, loss of muscle strength starts earlier than the mass and develops faster than the loss of mass. Accordingly, all updated consensus reports signified and integrated muscle strength/function as one of the two principal components of S in addition to the other principal component, LMM (4).

In practice, the clinicians already order abdominal CT/MRI for routine work-up, and this allows evaluating the regional skeletal muscle mass. Hence, recently similar studies have been carried out to examine if S is independently related to survival and other outcomes in cancer patients (5–9). Overall, in these studies, S was not associated with OS when considered as the presence of LMM. However, S was associated with OS when it was defined as the simultaneous presence of LMM and muscle strength/function. In a very recent consensus report on the definition of S, it has been signified that the studies on secondary S ignore the integration of impaired muscle strength/function into the diagnosis of S, albeit its current definition. Accordingly, they recommended that S studies in cancer as well should integrate low muscle strength/function in the definition of S in these studies (10). We suggest that if the authors would have integrated the presence of low muscle strength into the definition of S, they would find much higher association between OS and S, emphasizing the unique importance of S in the prognosis and assessment of the participants.

Why sarcopenia defined as LMM fails to correlate with survival in the majority of the studies, compared to when it is defined as loss of muscle strength and/or LMM + muscle strength, deserves attention and may be due to various factors. First, muscle strength (i.e., handgrip strength) has been shown to predict mortality and was associated with not only body composition changes but also nutritional status, inflammation, quality of life, and functionality as well, in multiple chronic diseases and advanced cancer patients (5, 11). Muscle strength may also capture important aspects of the adverse pathophysiological processes associated with cancer and aging (11). As an example, primary and secondary hypogonadism is seen as a complication in male cancer patients and is a well-known triggering factor for loss of muscle strength (12). The downregulation of IGF-1 expression in skeletal muscle might also be one of the factors playing an important role in the development of muscular atrophy in cancer patients, such as in those treated with chemotherapy (13). These strength-related hormonal factors, i.e., testosterone (14) and insulin-like growth factor-I (15, 16), which decline with the presence of cancer and muscle strength, might explain why strength appears to be such a powerful marker of risk. Furthermore, the loss of motor neurons with aging results in an increase in the size of the remaining motor units, but with greater preservation of type 1 fibers, resulting in preservation of mass with relatively fewer type 2 fibers and thus lower strength (17). This point may explain the better association of muscle strength than that of muscle mass in cancer patients, particularly in older ones.

In conclusion, we propose that clinicians should concentrate on muscle strength/function as well while studying sarcopenia and its association with cancer and treatment-related outcomes.

## Author Contributions

ZB: Writing—Original Draft Preparation and Editing. GB: Review and Editing. All authors contributed to the article and approved the submitted version.

## Conflict of Interest

The authors declare that the research was conducted in the absence of any commercial or financial relationships that could be construed as a potential conflict of interest.

## Publisher’s Note

All claims expressed in this article are solely those of the authors and do not necessarily represent those of their affiliated organizations, or those of the publisher, the editors and the reviewers. Any product that may be evaluated in this article, or claim that may be made by its manufacturer, is not guaranteed or endorsed by the publisher.
